# Role of keratan sulfate expression in human pancreatic cancer malignancy

**DOI:** 10.1038/s41598-019-46046-6

**Published:** 2019-07-04

**Authors:** Premila D. Leiphrakpam, Prathamesh P. Patil, Neeley Remmers, Benjamin Swanson, Paul M. Grandgenett, Fang Qiu, Fang Yu, Prakash Radhakrishnan

**Affiliations:** 1 Eppley Institute for Research in Cancer and Allied Diseases, Fred & Pamela Buffett Cancer Center, University of Nebraska Medical Center, Omaha, NE USA; 2 College of Medicine, University of Nebraska Medical Center, Omaha, NE USA; 3 Department of Pathology and Microbiology, University of Nebraska Medical Center, Omaha, NE USA; 4 College of Public Health, Biostatistics, University of Nebraska Medical Center, Omaha, NE USA

**Keywords:** Pancreatic cancer, Glycobiology

## Abstract

Keratan sulfate (KS) is a sulfated linear polymer of N-acetyllactosamine. Proteoglycans carrying keratan sulfate epitopes were majorly observed in cornea, cartilage and brain; and mainly involved in embryonic development, cornea transparency, and wound healing process. Recently, expression of KS in cancer has been shown to be highly associated with advanced tumor grade and poor prognosis. Therefore, we aimed to identify the expression of KS epitope in human pancreatic cancer primary and metastatic tumor lesions. Immunohistochemical analysis of KS expression was performed on primary pancreatic tumors and metastatic tissues. We observed an increased expression of KS epitope on primary tumor tissues compared to uninvolved normal and tumor stroma; and is associated with worse overall survival. Moreover, lung metastatic tumors show a higher-level expression of KS compared to primary tumors. Interestingly, KS biosynthesis specific glycosyltransferases expression was differentially regulated in metastatic pancreatic tumors. Taken together, these results indicate that aberrant expression of KS is predictive of pancreatic cancer progression and metastasis and may serve as a novel prognostic biomarker for pancreatic cancer.

## Introduction

Keratan Sulfate (KS) is a glycosaminoglycan (GAG) first detected in bovine cornea^1^. KS is widely distributed in both extracellular matrix (ECM) of cornea, bone, cartilage, brain, and on surface of the epithelial tissues^2,3^. Structurally, KS consists of a linear polymer of N-acetyllactosamine backbone of Galβ1-4GlcNacβ1-3 that has a sulfate moiety at C-6 positions of galactose (Gal) and N-acetylglucosamine (GlcNAc)^2,3^. KS biosynthesis occurs in two stages: production of GlcNAc-sulfated poly-N-acetyllactosamine chain by the sequential action of β1,3-N-aceltylglucosaminyltransferase 7 (β3GnT7), N-acetylglucosaminyl-6-sulfotransferase (GlcNAc6ST) and β1,4-galactosyltranferase 4 (β4GalT4); and production of highly sulfated KS by galactosyl-6-sulfotransferase (Gal6ST)^3–5^. In tissues, KS chains can be either N- or O-linked to the proteoglycan core protein. KS proteoglycans (KSPGs) with the family of small leucine-rich ECM PGs (keratocan, lumican, mimican, fibromodulin, osteomodulin and osteoadherin) are mainly composed of KS chains, whereas aggrecan with large chondroitin sulfate composition contains few KS chains^2,3^. In addition, epithelial mucin (MUC1)^6^ and an isoform of CD44^7^ have KS chains in their structure. These KSPGs are widely distributed in tissues and have diverse range of functions^2^.

KS plays an important role in corneal transparency, developmental biology, cell signaling, adhesion, and migration^2,8,9^. KS also plays a role in regulation of inflammation and has been reported to have potential in the treatment of inflammatory conditions such as rheumatoid arthritis and Chronic Obstructive Pulmonary Disease (COPD)^9,10^. Dysregulation of KS biosynthesis has been associated with numerous diseased conditions, including macular degeneration and keratoconus^2,5^, amyotrophic lateral sclerosis^11^, Alzheimer’s diseases^12^, and mucopolysaccharidosis IV^13^. In addition, several studies have reported that aberrant expression of KS is highly associated with malignant conditions such as Burkitt’s lymphoma^14^, astrocytic tumors^15^, glioblastoma^16^, and in human embryonic cancer cells^17^. Miyamoto *et al*. (2011) reported a consistent expression of KS in carcinomas of the female genital tract and the absence of KS expression in carcinoma of gastrointestinal tract, liver, and urinary tract suggesting that KS might be a potential cancer biomarker for differential diagnosis of these cancers^18^. Furthermore, the study also reported that carcinomas of lung, mesothelium, mammary gland, thyroid, and pancreas express KS^18^. Addition of sulfate group on the KS side chains induces p38 MAPK and PI3K mediated anti-apoptotic signaling pathways in Burkitt’s lymphoma cells^14^. KS specific sulfotransferases (CHST) 2/6/7, which catalyze the transfer of a sulfate group to the GlcNAc residues of KS side chains have been shown to play a vital role in tumor malignancy^14–16^. Differential expression of specific β1,3-N-aceltylglucosaminyltransferases (β3GnTs) involved in KS biosynthesis have also been reported in cancers^19–21^. Moreover, KSPGs like lumican are also upregulated in several cancer tissues and cell lines including pancreatic cancer^22–27^. However, the functional importance of KS and its biosynthetic glycosyltransferases expression in primary and metastatic pancreatic tumors remains elusive.

Pancreatic cancer is the fourth most common cause of cancer related death in the United States mainly due to disseminated diseased conditions, and has a 8% five-years survival rate^28^. Of the multiple histologic subtypes described, pancreatic ductal carcinoma (PDAC) is the most common and deadliest form of pancreatic cancer. Therefore, understanding the underlying molecular changes and the potential molecules involved in disease progression is critical for early diagnosis and therapeutic intervention. In this study, we explored the expression levels of KS and its biosynthesis specific enzymes in human PDAC primary and metastatic tumors. We detected differential expression of KS associated with organ specific metastasis. We also observed a differential expression of KS specific glycosyltransferases between primary and metastatic tumors. In addition to this, we also observed the correlation of aberrant expression of KS with increase risk and reduced overall patients’ survival.

## Results

### Keratan sulfate expression in pancreatic cancer

**Figure 1:**
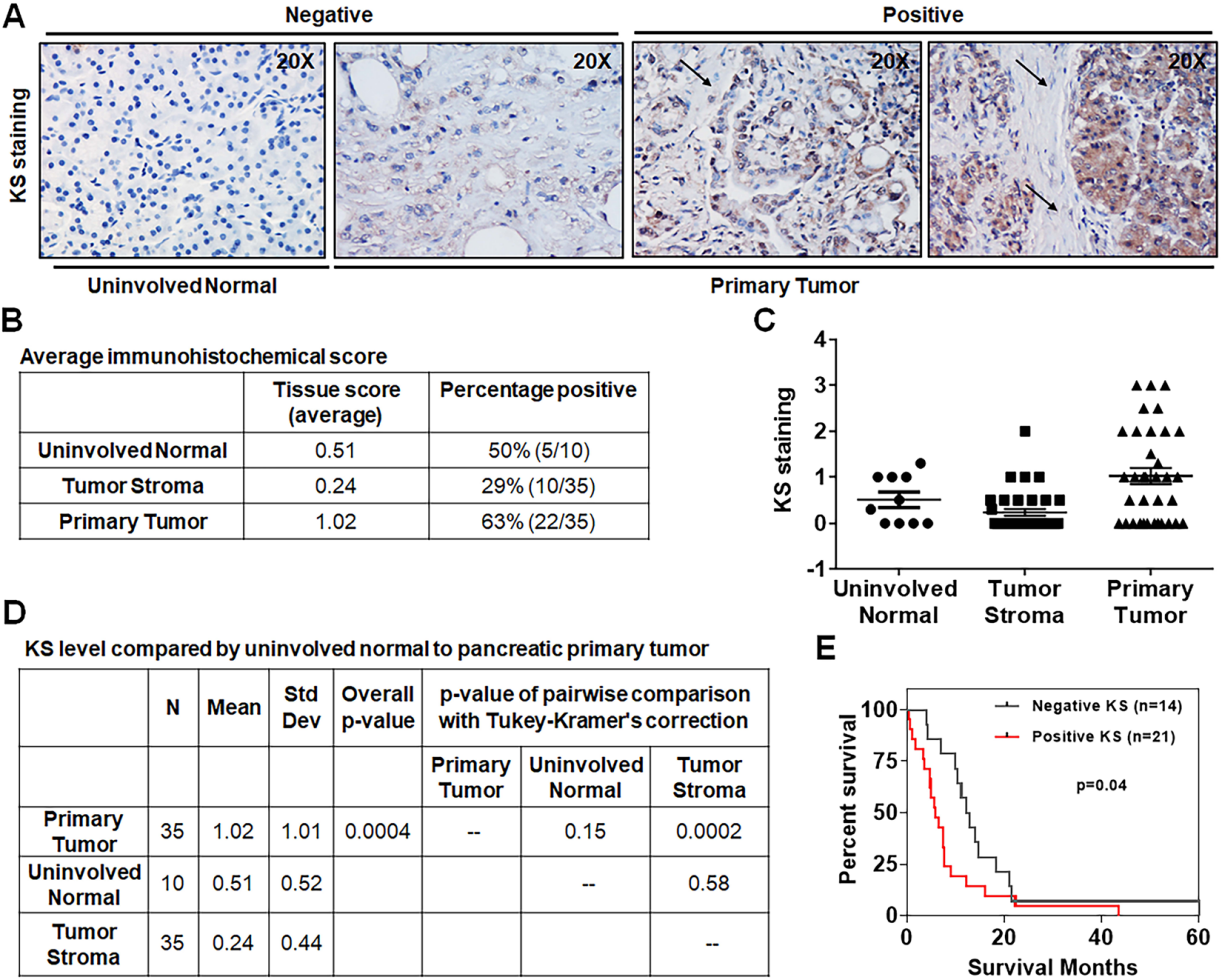
KS expression in primary pancreatic cancer. (**A**) Representative IHC staining of KS expression in primary tumor and corresponding uninvolved normal and tumor stromal tissues. Arrow represents tumor stroma. (**B**)The average IHC score of KS staining. (**C**,**D**) Statistically significant KS staining in primary tumor tissues compared to uninvolved normal and tumor stroma (p = 0.0004). (**E**) Kaplan Meier analysis of patients classified by positive and negative KS scores (n = 35), using the cutoff score of 0.05, showed correlation with overall patients’ survival (p = 0.04). A *p* value less than 0.05 considered statistically significant.

We examined the cellular distribution of keratan sulfate (KS) expression in pancreatic tumors immunohistochemically by using KS epitope specific antibody, I-22. For this, 35 pancreatic cancer patient samples were obtained through rapid autopsy program (RAP) as described in materials and methods section (Supplemental Table S1). Complete clinicopathologic features of these patients including age at diagnosis, gender, summary stage at diagnosis, and histological grade at autopsy are summarized in Table 1. In general, 16 (45.7%) are age >65 years and 19 (54.3%) are ≤65 years; 25 (71.4%) are male and 10 (28.6%) are female. Most of the patients are in stage III/IV with 28 (80%) and poorly or undifferentiated (G3/G4) histological grade with 21 (64%). H&E staining analysis on these tissues showed areas of tumor and stromal tissues (Supplemental Fig. S1A). Immunohistochemical analysis was performed in TMA samples of these primary tumor and uninvolved normal tissues for KS expression levels and Fig. 1A showed the pictorial representation of staining intensity. Heat map in Supplemental Fig. S1B provides a visual representation of the immunohistochemical score for relative KS expression levels on tumor cells of 35 patient’s primary tumor, 35 matched tumor stroma and 10 uninvolved normal tissues. Figure 1B shows the summary of staining for tissue sections that includes an average score for antigen expression in different normal and malignant cell types within the sections, and the number and percentage of patients that were positive for the indicated staining. Primary tumor cells showed a significantly increased expression pattern of KS side chains as compared to either uninvolved normal or tumor stromal tissues (p = 0.0004) (Fig. 1C,D). These findings indicate that KS is primarily localized in tumor cells. Clinicopathological parameters with comparisons to KS staining by groups in the 35 pancreatic cancer patients are summarized in Table 1. In general, positive KS expression was observed in 21 (60%) of 35 pancreatic tumor specimens and has significantly higher probability to be in stage III/IV compared to patients with KS negative staining (95 vs. 57.1, p value = 0.01). However, there is no association of KS staining with other clinicopathological features such as age at diagnosis, gender, and tumor grade (Table 1).

**Table 1:** Clinicopathological parameters with comparison to KS staining by groups in pancreatic cancer.

Clinicopathological Parameters^a^	Total	KS Expression		p value^†^
		Negative	Positive	
All cases	35	14	21	
Age at diagnosis				
>65	16 (45.7%)	6 (42.9%)	10 (47.6%)	0.78
≤65	19 (54.3%)	8 (57.1%)	11 (52.4%)	
Gender				
Male	25 (71.4%)	11 (78.6%)	14 (66.7%)	0.70
Female	10 (28.6%)	3 (21.4%)	7 (33.3%)	
Stage at diagnosis				
III/IV	28 (80%)	8 (57.1%)	20 (95.2%)	0.01
I/II	7 (20%)	6 (42.9%)	1 (4.8%)	
Grade*^b^				
G3-G4	21 (60%)	8 (66.7%)	13 (61.9%)	0.99
G1-G2	12 (40%)	4 (33.3%)	8 (38.1%)	

^a^All comparisons were made using fisher exact test, except the comparison of age (>65 vs ≤65), which was based on chi-square test.

*G1, well-differentiated; G2, moderately differentiated; G3, poorly differentiated; G4, undifferentiated.

^b^Two KS negative subjects have missing grade information.

^†^A p < 0.05 was regarded as statistically significant.

To evaluate the association of KS levels with pancreatic cancer patients’ prognosis, we performed survival analysis. Kaplan-Meier analysis showed that positive KS level is associated with a shorter median survival of 5.9 months compared to 12.5 months with negative KS expression and is significantly predictive of overall survival in patients (Fig. 1E). To further determine whether KS expression was an independent risk factor for patient survival prognosis, the Cox proportional hazard regression model was employed after accounting for the confounding effects of age at diagnosis, gender, stage at autopsy and histological grade (Table 2). The results showed that the risk of experiencing events in the KS positive group is 4.48 times the risk in the KS negative group after accounting the considered confounding factors (HR = 4.48; 95% CI = 1.51–13.34; p = 0.007). These findings further demonstrate the association of KS positive level with worst patient prognosis.

**Table 2:** Cox proportional hazard regression model for predictive factors of overall survival in patients with pancreatic cancer.

Clinicopathological Parameters	Hazard Ratio (HR)	95% Confidence Interval (CI)	p value^†^
Age at diagnosis (>65 vs ≤65)	1.01	0.46–2.20	0.98
Gender (Female vs Male)	0.20	0.07–0.59	0.003
Summary stage at diagnosis (III/IV vs I/II)	0.57	0.17–1.91	0.36
Histological grade at autopsy* (G3-G4 vs G1-G2)	1.84	0.75–4.50	0.18
KS staining (Positive vs Negative)	4.48	1.51–13.34	0.007

*G1, well-differentiated; G2, moderately differentiated; G3, poorly differentiated; G4, undifferentiated.

^**†**^A p < 0.05 was regarded as statistically significant.

### KS expression in pancreatic cancer metastasis

**Figure 2:**
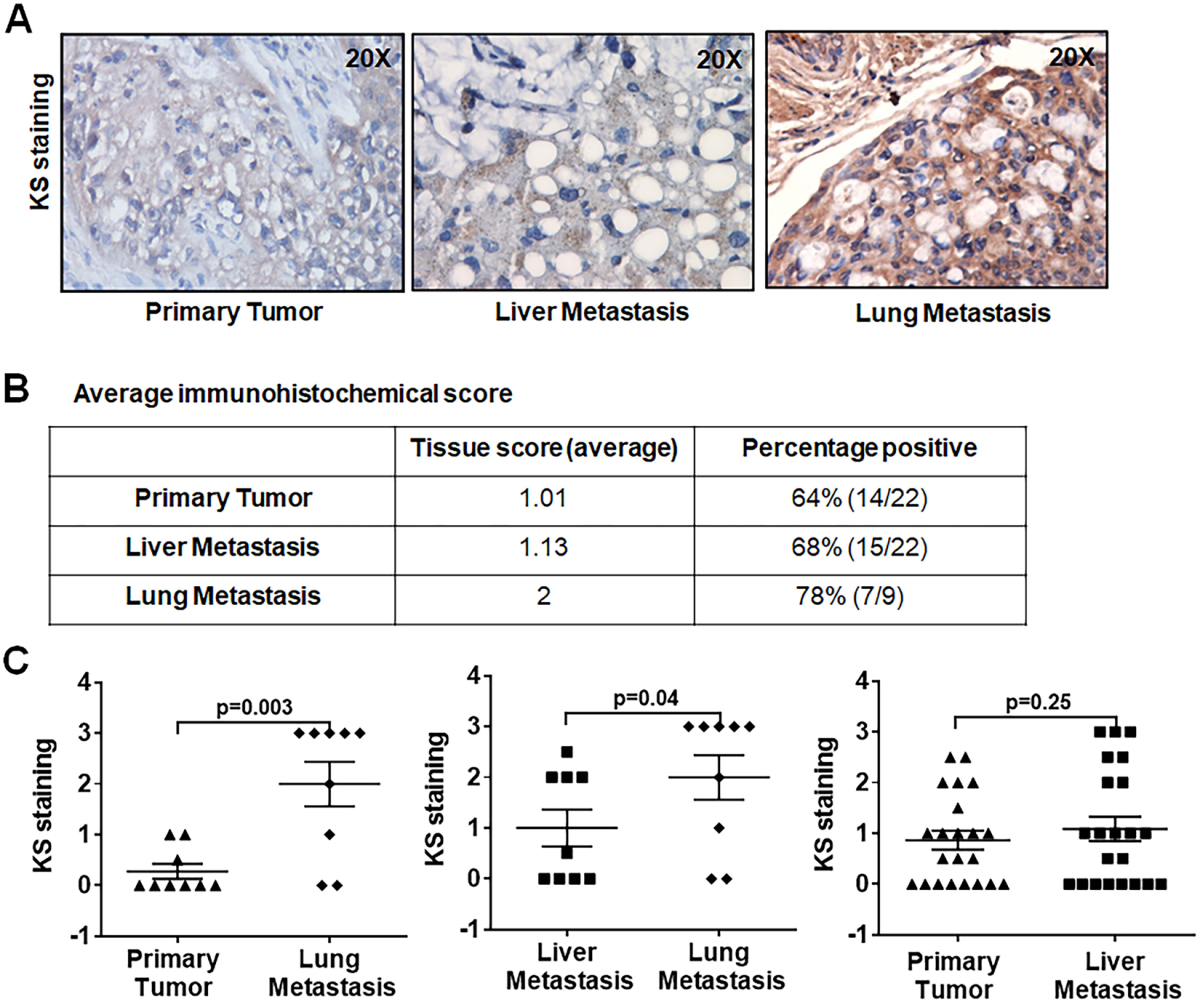
KS expression in liver and lung metastatic tumors. (**A**) Representative IHC staining of KS in primary tumors and matched sets of liver and lung metastases. (**B**) Average immunohistochemical score. (**C**) Comparisons of KS expression levels between primary tumor, lung, and liver metastatic tumor samples. A *p* value less than 0.05 considered statistically significant.

Pancreatic cancer metastasizes early and is the main cause of cancer related death. To further understand the correlation of KS expression with different organ specific metastasis of pancreatic cancers, we analyzed the expression pattern of KS between primary tumors and lymph node, liver and lung metastatic tumor tissues with IHC analysis. H&E staining were also performed on these tissues to localize areas of tumor and stromal tissues (Supplemental Fig. S1A). Figure 2A depicts KS staining in metastatic pancreatic tumor tissues. KS staining intensities were scored as described in materials and methods, and heat maps were generated to provide visual representation of score for relative KS expression level analyzed on tumor cells of primary tumor and metastatic tissues (Supplemental Fig. S2A). Figure 2B represents the summary of staining for tissue sections that includes an average score for antigen expression in different malignant cell types within the sections, and the number and percentage of patients that were positive for the KS staining. Both primary and metastatic deposits showed 64–78% KS staining in average. Lung metastatic deposits showed a significantly increased KS expression as compared to primary tumors (Fig. 2C left panel) and liver metastatic deposits (Fig. 2C middle panel). However, no significant difference was observed between primary tumors and liver metastatic deposits (Fig. 2C right panel). As expected, stromal tissues in lung and liver metastases have low KS expression compared to tumor cells (Supplemental Fig. S2B). These results indicate that higher levels of KS expression are associated with pancreatic cancer metastasis, preferably to lung tissues.

### mRNA expression profile of glycosyltransferase genes involved in KS biosynthesis

**Figure 3:**
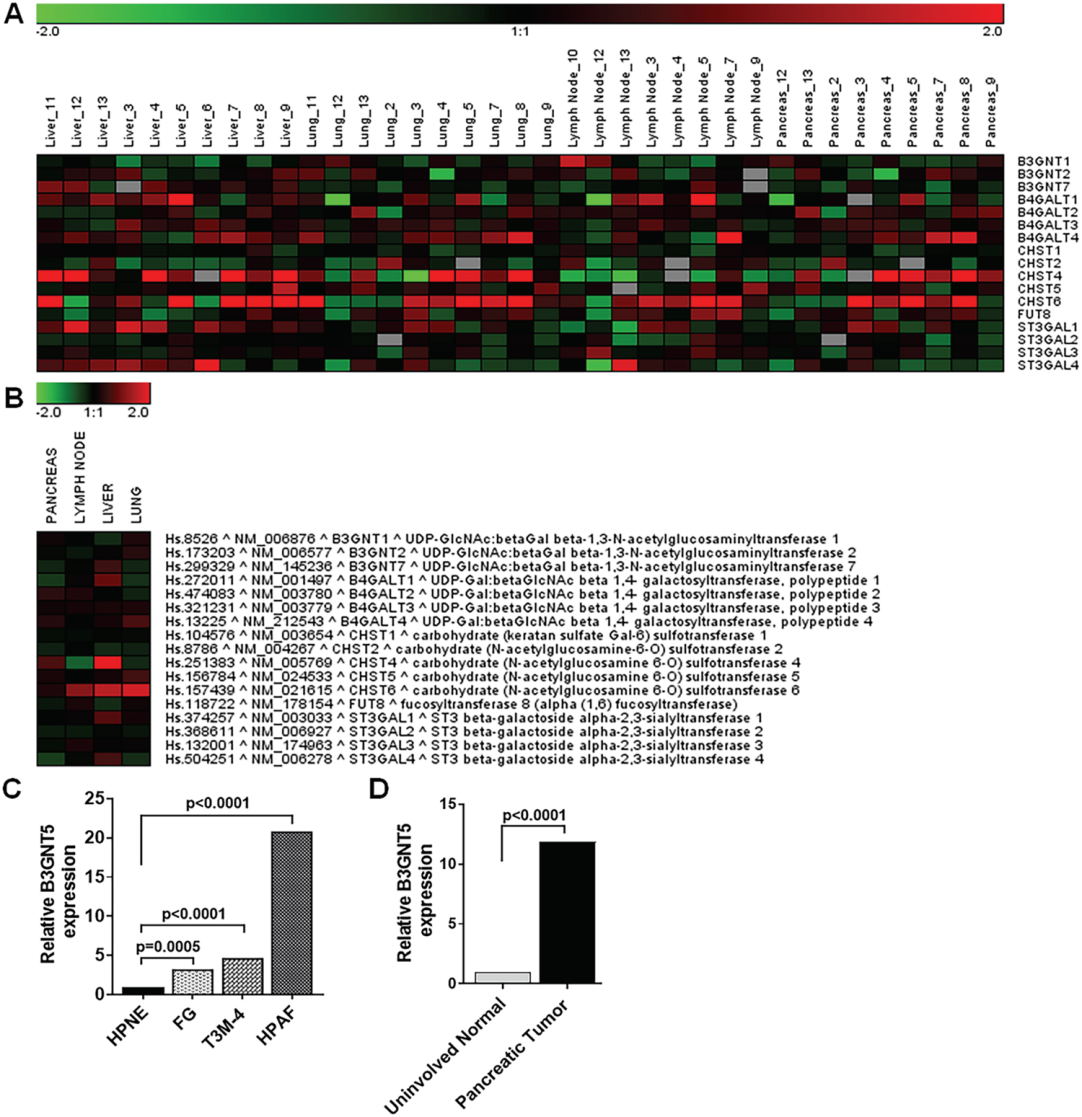
mRNA expression profiles of KS biosynthesis specific glycosyltransferase genes in pancreatic primary and metastatic tissues. The mRNA expression profiles from pancreatic cancer patients were analyzed on 80 Agilent human whole genome 4 × 44 K DNA microarrays. (**A**) The heatmap display the mean log2 fold change of the gene intensity between the samples from primary pancreatic cancer site or metastasis sites and the reference mRNA samples. (**B**) The heat map showed comparison of individual KS specific glycosyltransferase expression between the primary tumors, lymph node, liver and lung metastatic tumor tissues. (**C**) qRT-PCR analysis of *B3GNT5* gene expression in hTERT-HPNE, FG, T3M-4, and HPAF pancreatic cancer cell lines. (**D**) qRT-PCR analysis of *B3GNT5* gene expression in uninvolved normal pancreas and primary pancreatic tumors. A *p* value less than 0.05 considered statistically significant.

Dysregulation of glycosyltransferases expression that are involved in KS biosynthesis mediates aberrant KS expression in numerous pathophysiological conditions including cancers^2,9^. Increased sulfation of KS has been shown to correlate with progression in colorectal cancer, astrocytomas, and female genital tract carcinoma^8,15,16^. To check the status of KS specific glycotransferases in metastatic pancreatic cancer, gene expression array analyses were performed on the RNA extracted from RAP pancreatic primary tumor, metastatic tumor, and pancreatic normal tissues (Supplemental Table S2)^29^. 17 glycogenes (*B3GNT1-2/7, B4GALT1-4, CHST1-2/4-6, FUT8, and ST3GAL1-4*) involved in KS synthesis were measured in TMA samples of 12 primary tumors and corresponding lymph nodes, liver and lung metastatic deposits (Supplemental Table S3) and heat map generated as shown in Fig. 3A. The heat map showed differential expression of these glycogenes between primary tumors and metastatic deposits. Figure 3B represents the overall comparison of these genes in primary tumors and metastatic deposits.

**Figure 4:**
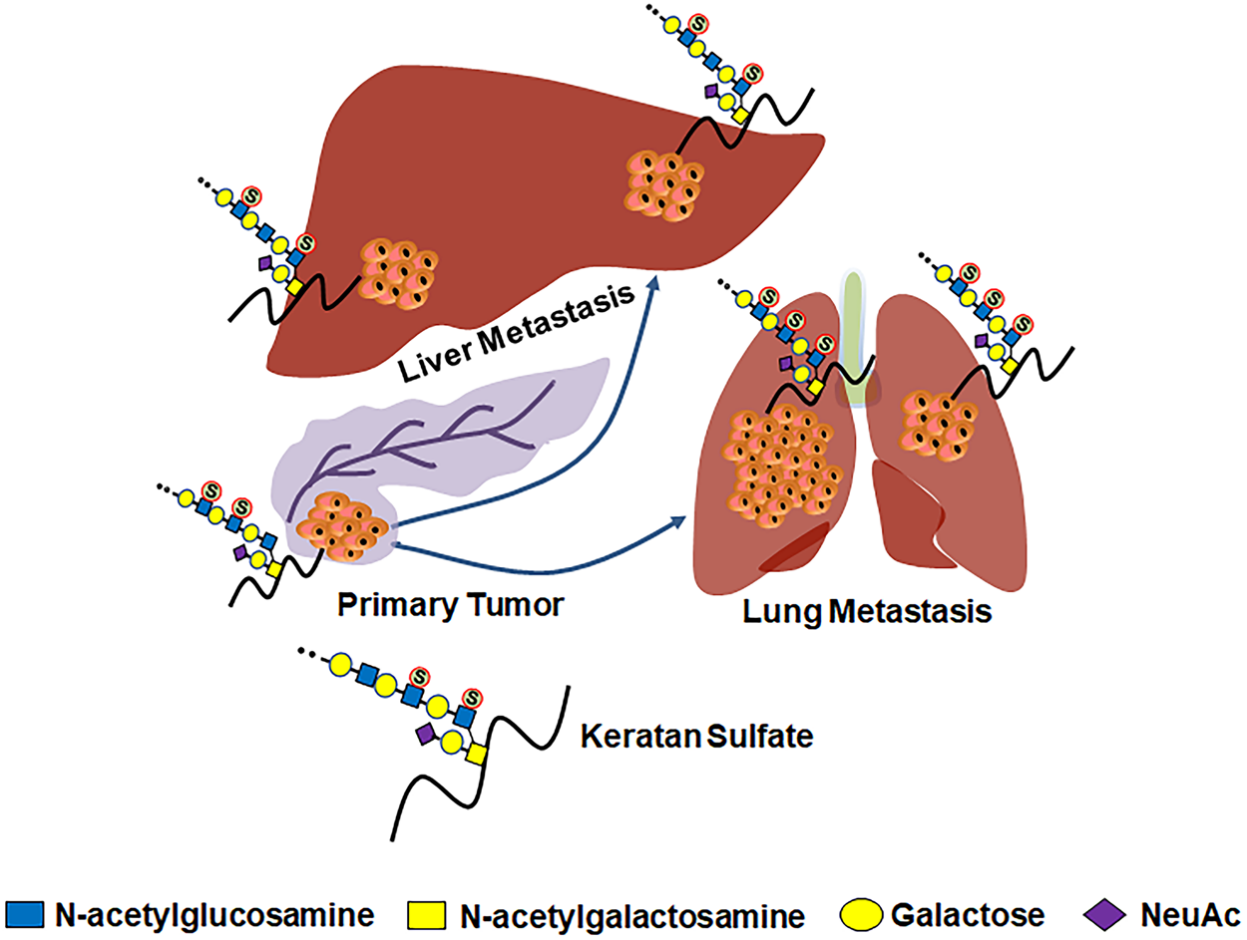
Proposed schematic diagram shows the role of aberrantly glycosylated KS in primary and metastatic pancreatic tumors.

Of the different genes encoding β3GnT enzyme, we examined the expression of *B3GNT1-2* and *B3GNT7* genes which showed an increase and decrease expressions, respectively, in lung metastatic tissues compared to primary tumor tissues (Fig. 3B). Among the genes encoding sulfotransferase (CHST) enzyme, *CHST6* gene expression was significantly upregulated in metastatic tissue deposits compare to primary tumors; whereas *CHST4* and *CHST5* gene expressions were increased in liver and lung metastatic deposits, respectively (Fig. 3B). These findings are similar to previous reports that showed association of increased sulfation of KS with tumorigenesis; and indicate that altered KS sulfation are partly involved in aberrant expression of KS epitopes in pancreatic cancer. In addition, genes encoding β4GalT and ST3Gal enzymes also showed differential expression between primary tumors and metastatic deposits (Fig. 3B). Moreover, PCR analysis showed significantly increased expression of *B3GNT5* mRNA in highly metastatic FG, T3M-4 and HPAF pancreatic cancer cell lines compared to HPNE (hTERT-immortalized human pancreatic epithelial nestin-expressing) cell line (Fig. 3C). As expected, human pancreatic tumor tissue has high *B3GNT5* mRNA expression compared to uninvolved normal tissues (Fig. 3D). These findings suggest that differential expression of these enzymes contributes to aberrant expression of KS epitopes in metastatic tumors. Altogether, these results demonstrate that differential expression of KS biosynthesis specific glycosyltransferases enhances the production of aberrantly glycosylated/sulfated KS side chains in primary and metastatic pancreatic cancer (Fig. 4).

## Discussion

KS is a major GAG shown to play an important role in developmental and physiological processes including tissue hydration, cell signaling and migration^2,5^. KS is formed through the elongation of N- or O-glycans attached covalently to scaffolds proteins^2^, and KS proteoglycans (KSPG) are found in numerous tissues^8^. Aberrant expression of KS results in numerous pathophysiological conditions including cancers^9^. Recent studies have shown that addition of sulfate group on the KS side chains induce p38 MAPK and PI3K mediated anti-apoptotic signaling pathways in cancer cells^14^. However, little is known about the correlation of KS expression with pancreatic cancer. The study presented here investigated the potentials of KS GAG as a non-invasive prognostic and metastatic predictive biomarker in patients with pancreatic cancer. We showed for the first time the correlation of aberrant KS expression with pancreatic cancer progression and metastasis and with worse overall patient survival status.

Experiments performed in a cohort of 35 human pancreatic cancers patients demonstrate a significant increase in KS expression levels in tumor cells than in uninvolved normal cells or tumor stroma. Moreover, KS expression levels were correlated with pancreatic cancer progression with significantly higher levels in patients with stage III/IV than in stage I/II. Interestingly, positive KS levels were significantly predictive of overall survival; and the Cox proportional hazard regression analysis showed patients with KS positive group has 4.48 times the risk of experiencing events compare to KS negative group. In addition, KS expression levels were upregulated in lung metastasis as compared to matched sets of primary tumors and / or liver metastases. Therefore, the study reported here clearly demonstrate that KS expression level is highly correlated with pancreatic cancer progression and metastasis as well as with worst patients’ survival status. More than 91% (32 out of 35) of these pancreatic cancer patients (all patients had metastatic spread) were given different regimen of chemotherapeutic drugs during the course of disease progression and did not show any statistical significance on KS expression (Supplemental Table S1). Maybe increase in sample size will provide a better representation in the correlation of patient clinicopathological status and overall survival with KS expression level, and could be explored in the future studies.

Malignant cells have more branched complex N-linked glycans, higher number of O-linked glycans, and mostly truncated versions designated as Tn, STn, and T antigens when compared to their normal counterparts leading to alteration of their carbohydrate chains^30,31^. These features are mainly caused by genetic and epigenetic dysregulation of glycosyltransferase genes and the tumor microenvironment, which results in increased cell proliferation, cell cycle progression, EMT, angiogenesis, and immune evasion^32^. Of these aberrant glycophenotypes, immature truncated O-glycan structures Tn and STn are the most common phenotype. These truncated O-glycans are present virtually in all types of epithelial cancer cells and numerous premalignant epithelial lesions^31,33^ and have been reported to be significantly correlated with worst prognosis^34^. Previously, we have reported that aberrant O-glycophenotype directly induces oncogenic features with enhanced cell growth and invasion in pancreatic cancer cells, and is critical during tumorigenesis^35^. Interestingly, studies have shown that O-glycopeptide epitopes with truncated O-glycan and a short peptide sequence have cancer-specific antigens properties and can be targeted by drug-loaded antibodies and T cell-engaging immunotherapies^36,37^. Furthermore, complex-type N-linked glycans with increased β1–6 branching has also been linked to cancer growth and metastasis^38^. *B3GNT2/5* genes encoding β3GnT enzyme, one of the critical glycosyltransferases involved in this process has been shown to be associated with cancer cell migration, invasion, and metastasis in cancers^39,40^, however, β3GnT7 enzyme decreases cancer cell motility and invasion^19,20^.

To understand the regulatory relationship between KS specific glycosyltransferases and KS epitope expression in metastatic pancreatic tumors, we evaluated the relative expression level of genes encoding KS biosynthesis specific glycosyltransferases in the primary and metastatic tumor tissues. Heat map in Fig. 3A demonstrated that the differential expression of these glycogenes in matched primary tumors and metastatic tissue deposits indicating these genes play a potential role in the differential expression of KS in pancreatic cancer. Previous studies have shown that an increased expression of highly sulfated KS was associated with the pathogenesis of several cancers^2,9^. As expected, expression of *CHST5-6 genes* encoding sulfotransferase enzyme are increased in lung metastasis when compared to primary tumor (Fig. 3B), and this finding correlates with higher KS side chain expression in lung metastatic deposits. Similar results were observed with *B3GNT1-2 genes* which encode β3GnT enzyme. In addition, mRNA expression of *B3GNT5 gene* was significantly increased in highly metastatic pancreatic cell lines and human pancreatic tumor tissues (Fig. 3C,D). In contrast, *B3GNT7 gene* expression remains constant between primary tumor and lung metastasis and is increased in liver metastasis (Fig. 3B). β3GnT is one of the key enzymes responsible for the synthesis of KS GAG; suppression of *B3GNT7 gene*^20^ and overexpression of *B3GNT2/5 genes*^40,41^ are associated with tumorigenesis of human cancer cells. We anticipated that increased *B3GNT7 gene* expression in liver metastasis compared to either primary tumor or lung metastatic tissues could be caused by either epigenetic or altered transcriptional regulatory mechanisms. Therefore, differential expression of these genes could contribute to the differential expression of KS in lung and liver metastatic deposits. Previously, studies have reported that sulfated GAGs modulate cancer cell differentiation, adhesion, migration as well as cancer cell interaction with ECM^42^. The findings in this study also suggest that increased sulfated KS levels are highly correlated with pancreatic cancer progression and metastasis.

Alterations observed in the expression of KS specific glycosyltransferase genes might result from tumor cells mediated modulations of oncogenic and tumor suppressor activities. 95% of pancreatic cancers carry activating mutations in KRAS, and up to 70% have mutated p53 tumor suppressor genes^43–45^. Increased expression of the ras oncogene has been linked to induce the β3GnT5 expression^46,47^, which results in impaired epithelial cell contact inhibition and increased cellular motility leading to neoplastic transformation of cells^48^. B3GNT5 has also been association with radio-resistant in Small Cell Lung Cancer cells through Bcl-2 cell mediated cell survival mechanism and induction of epithelial to mesenchymal transition (EMT)^39^. In this study, we demonstrated that a higher-level expression of B3GNT5 mRNA in KRAS and p53 mutated highly metastatic FG, T3M-4, and HPAF pancreatic cancer cell lines (Fig. 3C). Further, B3GNT5 mRNA expression was also increased in human primary pancreatic tumors which carry activating KRAS mutations in approximately 93% of patient cohort (Fig. 3D and data not shown). Interestingly, gain of function mutation in B3GNT2 has been reported to be associated with increased tumorigenic potential in cancers^40^. Moreover, transfer of a sulfate residue to position 6 of GlcNAc residues in KS by CHST2/6/7 has been reported to activate p38 MAPK-PI3K cell survival signaling pathway axis and reduced radiation induced apoptosis in Burkitt’s Lymphoma cells^14^. Therefore, it is predicted that mutations might contribute to the differential expression of KS specific glycosyltransferase genes and aberrant sulfation of KS, thereby leading to pancreatic cancer growth and metastasis. Further studies are necessary to delineate the regulation of differential glycosyltransferase gene expression in pancreatic cancer lesions leading to highly sulfated KS levels and increase pancreatic cancer tumorigenic potential.

## Conclusions

This study revealed a higher-level expression of KS in primary pancreatic tumor, and even increased in lung metastatic deposits and also associated with worse patient survival. The increase in KS expression levels were associated with the differential expression of its biosynthesis enzymes, mainly sulfotransferases. Overall, the results of this study support the concept that aberrant expression of KS contributes to aggressive nature of pancreatic tumors and their early metastases (Fig. 4) and could be used as a prognostic biomarker for pancreatic cancer. Further studies are warranted to investigate the mechanism by which KS enhances the malignant potentials of pancreatic cancer.

## Materials and Methods

### Patients

Human pancreatic tumors, metastatic lesions, and uninvolved normal tissue specimens from de-identified pancreatic cancer patients (n = 35) were obtained with consent and Institutional Review Board approval from University of Nebraska Medical Center’s Tissue Bank through the Rapid Autopsy Pancreatic (RAP) program. Of these, 32 patients (>91%) received different combinations of chemotherapy (Supplemental Table 1).

### Tissue microarray (TMA)

Formalin fixed tissues from RAP program are embedded in paraffin blocks and tissue microarrays (TMA) were prepared from these tissue sections using 2- to 2.5- mm punch. The arrays contained cores of matched normal and tumor tissues. Once completed, TMA blocks are cut into 4-micron thick sections with a microtome and mounted on charged slides.

### Immunohistochemistry

All immunohistochemistry analyses were performed using Dako EnVision kits (Dako, Glostrup, Denmark). Keratan sulfate (KS) specific mouse monoclonal antibody (I-22) was obtained from Developmental Studies Hybridoma Bank, Iowa University. Slides with serial sections of tissue microarrays were stained using KS mouse monoclonal antibody with standard IHC procedures. Briefly, tissues were deparaffinized in histoclear and rehydrated in descending grades of ethanol followed by antigen retrieval using an alkaline citrate buffer and microwave treatment. Endogenous peroxidase activity was quenched with 3% hydrogen peroxide and slides were blocked with 5% BSA. Following incubation with primary (1:100 dilution) and secondary antibodies substrate chromogen 3,3′-diaminobenzidine (DAB) was added followed by counterstaining with Harris hematoxylin and dehydration with alcohol gradients ending with xylene. Primary antibody concentrations and incubation conditions were optimized using positive control tissues. Specimens were processed on the same day to eliminate any variability in conditions. Whole slides scanning was performed using Ventana’s Coreo Au Slide Scanner in UNMC Tissue Science Facility.

### RNA isolation and microarray hybridization

We have utilized human whole genome 4 × 44 K DNA microarrays data from UNMC RAP tissue specimens (Supplemental Table S2)^29^. The data are publicly available in Gene Expression Omnibus database (http://www.ncbi.nlm.nih.gov/ geo/query/acc.cgi?acc = GSE21501). The two color Agilent arrays were used to quantify the relative gene expressions between samples from the metastasis sites or primary cancer sites versus the reference human mRNA samples (Stratagene). The array data were preprocessed by filtering the data to eliminate poor quality spots or genes that did not have mean intensity greater than 10 for either channel in at least 70% of the experiments. Lowess normalization were used to normalize the filtered data, and log2 ratio between the metastasis sites or primary cancer vs the reference mRNA samples were calculated. The ratios with at least 30 for the normalized raw intensity from both channels were included for further heat map analysis, which provide a visual comparison in the gene expression between metastasis site and primary pancreatic cancer.

### Cell lines

HPNE cell line is an hTERT (active telomerase) immortalized human pancreatic Nestin expressing (HPNE) ductal progenitor cell line is undifferentiated and non-tumorigenic^49^. FG cells are fast growing clone established from the Colo-357 pancreatic ductal adenocarcinoma cell line^50^. T3M4, a human carcinoembryonic antigen (CEA)-producing cell line was established from a primary PDAC transplanted into nude mice and then cultured^51^. HPAF cell line was originally derived from the ascetic fluid of patient with pancreatic cancer^52^. All cell lines were maintained in DMEM (Dulbecco’s Modified Eagle Medium) (Hyclone Laboratories, Logan, UT) supplemented with 10% heat deactivated fetal bovine serum (Sigma, St. Louis, MO), penicillin G (100 U/mL), and streptomycin (100 µg/mL) (Corning Life Sciences, Corning, NY) at 37 °C with 5% CO2 in a humidified atmosphere as described previously^35^.

### Quantitative Real-Time PCR

Quantitative Real-Time PCR (qRT-PCR) analysis was performed as described previously^53^. Briefly, total cellular RNAs were isolated from HPNE, FG, T3M-4 and HPAF pancreatic cancer cell lines and pancreatic tumor and uninvolved normal tissues using TRI reagent (Molecular Research Center, Inc, USA) as per the manufacturer’s protocol. RNA sample integrity was measured by Nanodrop spectrophotometry and Aligent Bioanalyzer and cDNA was synthesized from total cellular RNAs using Verso^TM^ cDNA kit (ABgene, UK). QRT-PCR was performed on a Master cycler *realplex*^2^ (Eppendorf AG, Hamburg) using SYBER Green PCR kit (Takara Bio Inc, USA). Forward and reverse primers, 5′-TATTCACATGGGAGAGCCGCATGA-3′ and 5′-TGCGGAAGAATGGAACACCCTGTA-3′ respectively, were used for selective amplification of *B3GNT5*. The data were analyzed using Eppendorf realplex software, version 1.5 (Eppendorf). Amplification of GAPDH (forward primer, 5′-TCGACAGTCAGCCGCATCTTCTTT-3′ and reverse primer, 5′-ACCAAATCCGTTGACTCCGACCTT-3′) was used to normalize the samples for comparison.

### Tissue analysis

Histological sections were annotated by two independent pathologists. Relative KS antigen expression level was semi-quantified based on percentage of cells staining positive for KS antigen. A scale of 0–3 was used to indicate relative percentage of cells positive with 0 being no detectable expression and 3 indicating ≥67% of the total cell population expressed KS antigen. Discrepancies between two pathologists were rare and were resolved by averaging the results. 2–4 sections from each tumor were present on tissue microarrays and the final score for each sample was based on the mean score from all sections stained, to best represent the spectrum of tumor heterogeneity. Staining intensity scores were converted into heat maps for better visualization.

### Statistical analysis

Statistical analysis was performed using SAS software (Version 9.4, SAS Institute Inc., Cary, NC, USA) and Graph Prism 7 software. Comparison between immunohistochemical scores of different groups in the matched autopsy samples were analyzed using paired t-tests between two groups or linear mixed models across more than two groups. Post-hoc tests with Turkey’s method were used for multiple comparisons when appropriate. Chi-square tests or Fisher’s exact tests were used to compare the categorical clinicopathological parameters between KS staining positive and KS staining negative groups. Kaplan-Meier plot was used to describe overall survival between groups and log-rank test was used for comparison. The Cox proportional hazard regression model was utilized to assess the association between KS staining with overall survival after accounting for the confounding effects of age, gender, stage, and grade.

## Supplementary information

Supplemental Information
