# Supplementary material for: Role of keratan sulfate expression in human pancreatic cancer malignancy
Source: Sci Rep. 2019 Jul 4;9:9665. doi: 10.1038/s41598-019-46046-6 (PMC6609602; doi:10.1038/s41598-019-46046-6)
Supplement: Supplementary file 1 — Supplemental Information [file 41598_2019_46046_MOESM1_ESM.docx]

**Role of keratan sulfate expression in human pancreatic cancer malignancy**

Premila D. Leiphrapkam^1^, Prathamesh P. Patil^1^, Neeley Remmers^1,2^, Ben Swanson^1,3^, Paul M. Grandgenett^1^, Fang Yu^4^, Fang Qiu^4^ and Prakash Radhakrishnan^1,3*^

^1^Eppley Institute for Research in Cancer and Allied Diseases, Fred & Pamela Buffett Cancer Center, University of Nebraska Medical Center, Omaha, NE, USA.

^2^College of Medicine, University of Nebraska Medical Center, Omaha, NE, USA.

^3^Department of Pathology and Microbiology, University of Nebraska Medical Center, Omaha, NE, USA.

^4^College of Public Health, Biostatistics, University of Nebraska Medical Center, Omaha, NE, USA.

*Corresponding Author:

**Prakash Radhakrishnan**

Eppley Institute for Research in Cancer and Allied Diseases

Fred & Pamela Buffett Cancer Center

University of Nebraska Medical Center,

Omaha, NE, 68198-6805, USA

E-mail: [pradhakr@unmc.edu](mailto:pradhakr@unmc.edu)

Phone: 402-559-0845

**Supplemental Table S1: Rapid autopsy program (RAP) patients’ details**

| **RAP #** | **Age at diagnosis (years)** | **Gender** | **Summary stage at diagnosis** | **Chemotherapy** | **Histology grade at autopsy**  **(UNK=Unknown)** |
| --- | --- | --- | --- | --- | --- |
| 03 | 78 | F | IB | Yes | Moderate to poorly differentiated |
| 04 | 59 | M | III | Yes | Moderate to poorly differentiated |
| 05 | 65 | F | IV | Yes | Poorly differentiated |
| 06 | 62 | M | IV | Yes | Moderate to poorly differentiated |
| 07 | 71 | M | IV | Yes | Moderate to poorly differentiated |
| 08 | 72 | M | IV | No | Moderately differentiated |
| 09 | 69 | M | IV | Yes | Poorly differentiated |
| 12 | 82 | M | IV | Yes | Poor to undifferentiated |
| 16 | 70 | M | IV | Yes | Poor to undifferentiated |
| 17 | 63 | F | IIA | Yes | UNK |
| 18 | 82 | M | IV | Yes | UNK |
| 19 | 65 | M | IV | Yes | Poorly differentiated |
| 20 | 77 | M | IV | Yes | Poorly differentiated |
| 21 | 60 | F | IV | Yes | Poorly differentiated |
| 22 | 65 | F | III | Yes | Well differentiated |
| 23 | 76 | F | III | Yes | Poorly differentiated |
| 25 | 74 | M | IV | Yes | Poorly differentiated |
| 26 | 48 | M | IV | Yes | Poorly differentiated |
| 27 | 60 | M | IV | Yes | Well differentiated |
| 29 | 80 | F | IV | No | Moderate to poorly differentiated |
| 30 | 55 | M | IV | Yes | Poorly differentiated |
| 31 | 79 | M | IV | Yes | Moderately differentiated |
| 32 | 59 | M | IV | Yes | Moderate to poorly differentiated |
| 33 | 50 | M | IV | Yes | Moderate to poorly differentiated |
| 34 | 81 | M | IIB | No | Poorly differentiated |
| 35 | 62 | M | III | Yes | Moderate to poorly differentiated |
| 36 | 80 | F | IIB | Yes | Well to moderately differentiated |
| 40 | 62 | M | IV | Yes | Poorly differentiated |
| 44 | 58 | F | IIA | Yes | Moderately differentiated |
| 53 | 57 | M | IIB | Yes | Moderately differentiated |
| 54 | 68 | M | IV | Yes | Moderately differentiated |
| 55 | 55 | F | IV | Yes | Well differentiated |
| 56 | 74 | M | III | Yes | Moderately differentiated |
| 57 | 56 | M | IV | Yes | Moderately differentiated |
| 58 | 60 | M | IIB | Yes | Moderately differentiated |

**Supplemental Table S2: Tissue sections from pancreatic cancer patient autopsies utilized for gene expression arrays**

| **Subject by tissues** | | | | | |
| --- | --- | --- | --- | --- | --- |
| **RAP#** | **Tissue** | | | | |
|  | **Primary** | **Metastasis** | | |  |
|  | **Pancreas** | **Lymph node** | **Liver** | **Lung** | **Total** |
| 2 | 1 | 0 | 0 | 0 | **1** |
| 3 | 1 | 4 | 1 | 1 | **7** |
| 4 | 1 | 1 | 3 | 0 | **5** |
| 5 | 1 | 1 | 4 | 0 | **6** |
| 6 | 0 | 0 | 1 | 0 | **1** |
| 7 | 1 | 1 | 1 | 2 | **5** |
| 8 | 1 | 0 | 1 | 0 | **2** |
| 9 | 3 | 1 | 2 | 2 | **8** |
| 10 | 0 | 1 | 0 | 0 | **1** |
| 11 | 0 | 0 | 3 | 2 | **5** |
| 12 | 4 | 2 | 2 | 1 | **9** |
| 13 | 2 | 1 | 2 | 0 | **5** |
| **Total** | **15** | **12** | **20** | **8** | **55** |

**Supplemental Table S3. Gene expression profile of KS biosynthesis specific glycosyltransferase enzymes**

| **Sr.**  **No** | **Genes** | **Tissues*** | **RAP**  **10** | **RAP**  **11** | **RAP**  **12** | **RAP**  **13** | **RAP**  **2** | **RAP**  **3** | **RAP**  **4** | **RAP**  **5** | **RAP**  **6** | **RAP**  **7** | **RAP**  **8** | **RAP**  **9** | **Total** |
| --- | --- | --- | --- | --- | --- | --- | --- | --- | --- | --- | --- | --- | --- | --- | --- |
| **1** | **B3GNT1** | **Primary** |  |  | 0.61 | 0.16 | -0.2 | 0.16 | 0.24 | -0.47 |  | -0.6 | -0.4 | 0.44 | -0.01 |
|  |  | **LN** | 1.63 |  | 0.87 | -0.1 |  | -0.1 | -0.44 | -0.87 |  | -0.1 |  | 0.23 | 0.14 |
|  |  | **Liver** |  | -0.17 | -0.03 | -0.2 |  | -0.16 | -0.35 | -0.45 | -0.99 | -0.1 | -0.6 | -0.1 | -0.31 |
|  |  | **Lung** |  | 0.27 | 0.97 |  |  |  |  |  |  | 0.49 |  | 0.22 | 0.49 |
| **2** | **B3GNT2** | **Primary** |  |  | -0.54 | 0.65 | -0.01 | 0.65 | -1.5 | 0.08 |  | 0.73 | 0.4 | 0.08 | 0.06 |
|  |  | **LN** | 0.38 |  | -0.59 | 0.72 |  | 0.72 | -0.74 | 0.34 |  | 0.06 |  |  | 0.13 |
|  |  | **Liver** |  | -0.09 | 0.3 | 0.35 |  | 0.35 | -0.55 | -0.15 | -0.35 | 0.07 | 0.2 | 0.71 | 0.08 |
|  |  | **Lung** |  | 0.75 | -0.05 |  |  |  |  |  |  | -0.1 |  | 0.3 | 0.23 |
| **3** | **B3GNT7** | **Primary** |  |  | -0.37 | -0.2 | -0.65 | -0.15 | -0.18 | -0.08 |  | -0.8 | -0.1 | -0.3 | -0.3 |
|  |  | **LN** | -0.6 |  | -0.21 | -0 |  | -0.03 | 0.03 | 0.76 |  | 0.16 |  |  | 0.01 |
|  |  | **Liver** |  | 0.97 | 0.95 | -0.5 |  | -0.5 | 0.83 | 0.26 | 0.08 | 0.44 | 0.1 | 0.56 | 0.32 |
|  |  | **Lung** |  | -0.25 | -0.11 |  |  |  |  |  |  | -0.4 |  | -0.4 | -0.28 |
| **4** | **B4GALT1** | **Primary** |  |  | -1.25 | 0.03 | 0.09 | 0.03 | -0.28 | 1.2 |  | -0.8 | 0.3 | -0.5 | -0.13 |
|  |  | **LN** | -0.1 |  | -1.98 | 1.03 |  | 1.03 | 0.2 | 2.18 |  | -0.5 |  | 0.09 | 0.25 |
|  |  | **Liver** |  | 0.78 | 0.35 | 1.07 |  | 1.07 | 1.08 | 2.32 | 0.28 | -0.7 | 0.3 | 0.64 | 0.72 |
|  |  | **Lung** |  | 0.42 | -2.64 |  |  |  |  |  |  | -1.2 |  | -0.2 | -0.92 |
| **5** | **B4GALT2** | **Primary** |  |  | 0.05 | 1.12 | -1.05 | 1.12 | 0.36 | 0.23 |  | -0.3 | 0.7 | 0.87 | 0.34 |
|  |  | **LN** | 0.32 |  | 0.25 | 0.4 |  | 0.4 | 0.17 | 0.55 |  | 0.08 |  | 0.06 | 0.28 |
|  |  | **Liver** |  | -0.31 | -0.35 | -0.3 |  | -0.35 | 0.18 | 0.58 | -0.51 | -0.1 | 0.6 | 0.11 | -0.05 |
|  |  | **Lung** |  | -0.09 | -0.33 |  |  |  |  |  |  | -0.2 |  | -0.5 | -0.26 |
| **6** | **B4GALT3** | **Primary** |  |  | -0.21 | 0.17 | 0.44 | 0.17 | 0.56 | -0.34 |  | 0.29 | -0.1 | 0.27 | 0.14 |
|  |  | **LN** | -0.2 |  | -0.54 | 0.27 |  | 0.27 | 0.3 | -0.13 |  | 0.46 |  | -0.3 | 0.02 |
|  |  | **Liver** |  | 0.16 | -0.23 | -0.5 |  | -0.47 | 0.37 | 0.27 | 0.78 | 0.53 | -0 | 0.22 | 0.12 |
|  |  | **Lung** |  | 0.45 | -0.34 |  |  |  |  |  |  | 0.43 |  | 0.14 | 0.17 |
| **7** | **B4GALT4** | **Primary** |  |  | -0.38 | 0.32 | -0.22 | 0.32 | 0.84 | 0.54 |  | 1.53 | 1.8 | 0.18 | 0.54 |
|  |  | **LN** | -0.2 |  | -0.63 | 0.63 |  | 0.63 | -0.3 | -0.87 |  | 2.32 |  | -0 | 0.19 |
|  |  | **Liver** |  | 0.26 | 0.78 | 0.34 |  | 0.34 | -0.39 | -0.63 | 0.99 | 1.26 | 0.8 | 0.34 | 0.4 |
|  |  | **Lung** |  | 1.08 | -0.39 |  |  |  |  |  |  | 0.87 |  | 0.16 | 0.43 |
| **8** | **CHST1** | **Primary** |  |  | 0.017 | -0 | -0.28 | -0.02 | -0.57 | -0.32 |  | -0.1 | -0.1 | -0.1 | -0.16 |
|  |  | **LN** | 0.31 |  | -0.16 | 0.32 |  | 0.32 | -0.11 | -0.63 |  | 0.13 |  | 0.08 | 0.03 |
|  |  | **Liver** |  | 0.05 | 0.02 | 0.03 |  | 0.03 | -0.15 | -0.13 | -0.06 | -0 | 0.4 | -0.5 | -0.03 |
|  |  | **Lung** |  | -0.13 | -0.03 |  |  |  |  |  |  | -0 |  | -0.2 | -0.1 |
| **9** | **CHST2** | **Primary** |  |  | -0.52 | -0.6 | 1.1 | -0.62 | -0.21 |  |  | -0 | -0.8 | -0.1 | -0.21 |
|  |  | **LN** | 0.64 |  | -1.23 | -0.5 |  | -0.53 |  | 0.44 |  | 0.16 |  | 0.43 | -0.09 |
|  |  | **Liver** |  | -0.09 | -0.55 | 0.65 |  | 0.65 | -0.41 | -0.71 | -0.73 | -0.6 | 0.1 | -0.2 | -0.19 |
|  |  | **Lung** |  | 0.12 | -1.65 |  |  |  |  |  |  | -0.2 |  | -0.2 | -0.48 |
| **10** | **CHST4** | **Primary** |  |  | -0.86 | 1.18 | -1.09 | 1.18 | 2.38 | 1.86 |  | 1.3 | 2.2 | 1.11 | 1.03 |
|  |  | **LN** | -1.4 |  | -1.14 | -1.7 |  | -1.7 |  | -1.29 |  | 0.61 |  | -1.1 | -1.1 |
|  |  | **Liver** |  | 2.88 | 3.19 | 0.56 |  | 0.56 | 2.62 | 0.98 |  | 2.48 | 1 | 2.78 | 1.9 |
|  |  | **Lung** |  | 0.93 | -1.41 |  |  |  |  |  |  | 0.68 |  | -1.1 | -0.23 |
| **11** | **CHST5** | **Primary** |  |  | 0.24 | 0.79 | -0.13 | 0.79 | 0.13 | 0.53 |  | -0.7 | -0.1 | 0.29 | 0.21 |
|  |  | **LN** | -0.3 |  | 0.17 |  |  |  | -0.14 | 0.68 |  | 0.01 |  | 1.11 | 0.26 |
|  |  | **Liver** |  | -0.18 | 0.2 | -0.1 |  | -0.13 | 0.27 | 0.07 | 0.09 | -0.2 | -0 | 1.53 | 0.15 |
|  |  | **Lung** |  | -0.01 | 0.22 |  |  |  |  |  |  | -0.1 |  | 1.75 | 0.46 |
| **12** | **CHST6** | **Primary** |  |  | -0.56 | -0.8 | -0.74 | -0.81 | 1.36 | 2.93 |  | 1.06 | 2.3 | -0.5 | 0.47 |
|  |  | **LN** | 0.52 |  | -1.23 | 0.91 |  | 0.91 | 1.18 | 4.48 |  | 1.82 |  | 0.46 | 1.13 |
|  |  | **Liver** |  | 2.60 | -1.34 | 0.36 |  | 0.36 | -0.5 | 4.07 | -1.11 | 2.7 | 2.8 | 2.27 | 1.22 |
|  |  | **Lung** |  | 3.57 | -2 |  |  |  |  |  |  | 2.05 |  | 2.05 | 1.42 |
| **13** | **FUT8** | **Primary** |  |  | -0.99 | 0.42 | 0.18 | 0.42 | -0.62 | 0.18 |  | 0.46 | 0.7 | -0.3 | 0.05 |
|  |  | **LN** | 0.08 |  | -1.15 | 0.59 |  | 0.59 | -0.09 | 1.06 |  | 1.23 |  | -0.4 | 0.25 |
|  |  | **Liver** |  | -0.19 | -0.95 | -0.2 |  | -0.15 | -0.96 | -0.55 | -0.25 | 0.04 | 0.7 | 0.09 | -0.24 |
|  |  | **Lung** |  | 0.23 | -1.31 |  |  |  |  |  |  | 0.36 |  | -0.3 | -0.26 |
| **14** | **ST3GAL1** | **Primary** |  |  | -0.09 | -0.3 | 0.23 | -0.34 | 0.8 | -0.56 |  | 0.57 | -0 | -0.5 | -0.02 |
|  |  | **LN** | -0.9 |  | -0.22 | -1.4 |  | -1.36 | 0.66 | -0.36 |  | 0.08 |  | -0.3 | -0.47 |
|  |  | **Liver** |  | 0.68 | 1.83 | 0.46 |  | 0.46 | 1.37 | -0.18 | 1.13 | 0.66 | 0.2 | 0.61 | 0.72 |
|  |  | **Lung** |  | 0.32 | 0.33 |  |  |  |  |  |  | 0.25 |  | -0.5 | 0.1 |
| **15** | **ST3GAL2** | **Primary** |  |  | 0.03 | -0.1 |  | -0.12 | -0.07 | 0.14 |  | -0.9 | 0.1 | -0.6 | -0.19 |
|  |  | **LN** | 0.02 |  | 0.26 | -1.1 |  | -1.09 | -0.51 | 0.59 |  | 0.24 |  | -0.1 | -0.21 |
|  |  | **Liver** |  | -0.32 | -0.26 | -0.2 |  | -0.17 | 0.02 | 0.28 | -0.08 | -0 | 0 | 0.06 | -0.06 |
|  |  | **Lung** |  | 0.01 | -0.04 |  |  |  |  |  |  | -0.4 |  | -0.3 | -0.18 |
| **16** | **ST3GAL3** | **Primary** |  |  | -0.1 | 0.06 | -0.75 | 0.06 | 0 | 0.3 |  | -0.6 | 0 | -0.3 | -0.14 |
|  |  | **LN** | 0.26 |  | 1.07 | -0.2 |  | -0.15 | 0.16 | 0.69 |  | 0.19 |  | 0.26 | 0.29 |
|  |  | **Liver** |  | -0.08 | 0.39 | -0.1 |  | -0.13 | -0.28 | 0.33 | -0.09 | 0.12 | -0.2 | 0.17 | 0.01 |
|  |  | **Lung** |  | 0.24 | 0.59 |  |  |  |  |  |  | -0.5 |  | 0.01 | 0.09 |
| **17** | **ST3GAL4** | **Primary** |  |  | -1 | 0.5 | -0.05 | 0.5 | -0.32 | -0.73 |  | -0.6 | -0.4 | -0.6 | -0.29 |
|  |  | **LN** | -0.03 |  | -1.66 | 1.83 |  | 1.83 | 0.29 | -0.31 |  | -0.7 |  | -0.3 | 0.12 |
|  |  | **Liver** |  | 0.6 | 0.75 | 0.97 |  | 0.97 | 0.82 | 0.52 | 1.95 | -0.5 | 0.04 | -0.5 | 0.57 |
|  |  | **Lung** |  | 0 | -1.79 |  |  |  |  |  |  | -0.5 |  | -0.5 | -0.68 |
| *Primary, Pancreatic Primary Tumor; LN, Lymph Node Metastasis; Liver, Liver Metastasis; Lung, Lung Metastasis | | | | | | | | | | | | | | | |

**Supplemental Figure S1**

**
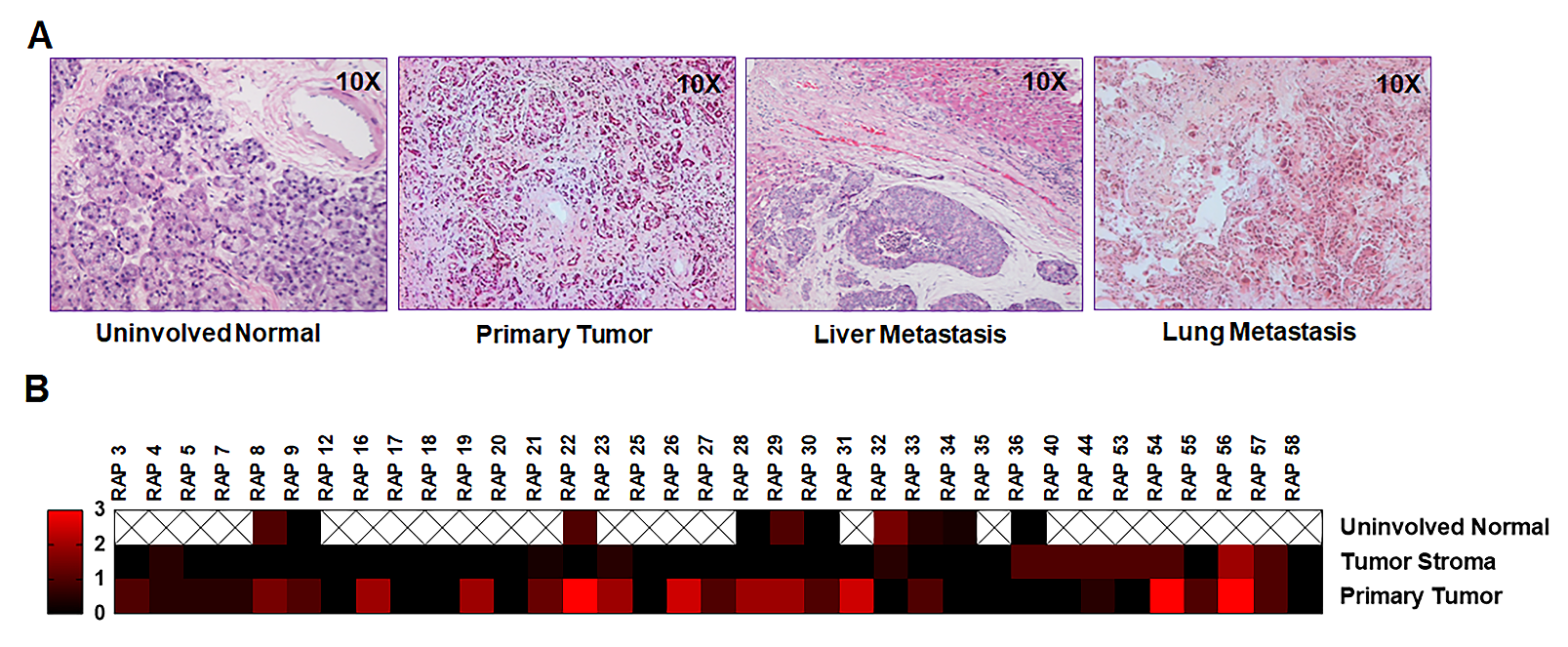
**

**Supplemental Figure S1. Hematoxylin and eosin staining and KS expression in primary pancreatic cancer.** (A) H&E staining of uninvolved normal pancreas, primary pancreatic tumor, liver and lung metastatic tissues. (B) Heat map of uninvolved normal pancreas, tumor stroma, and primary pancreatic tumor.

**Supplemental Figure S2**


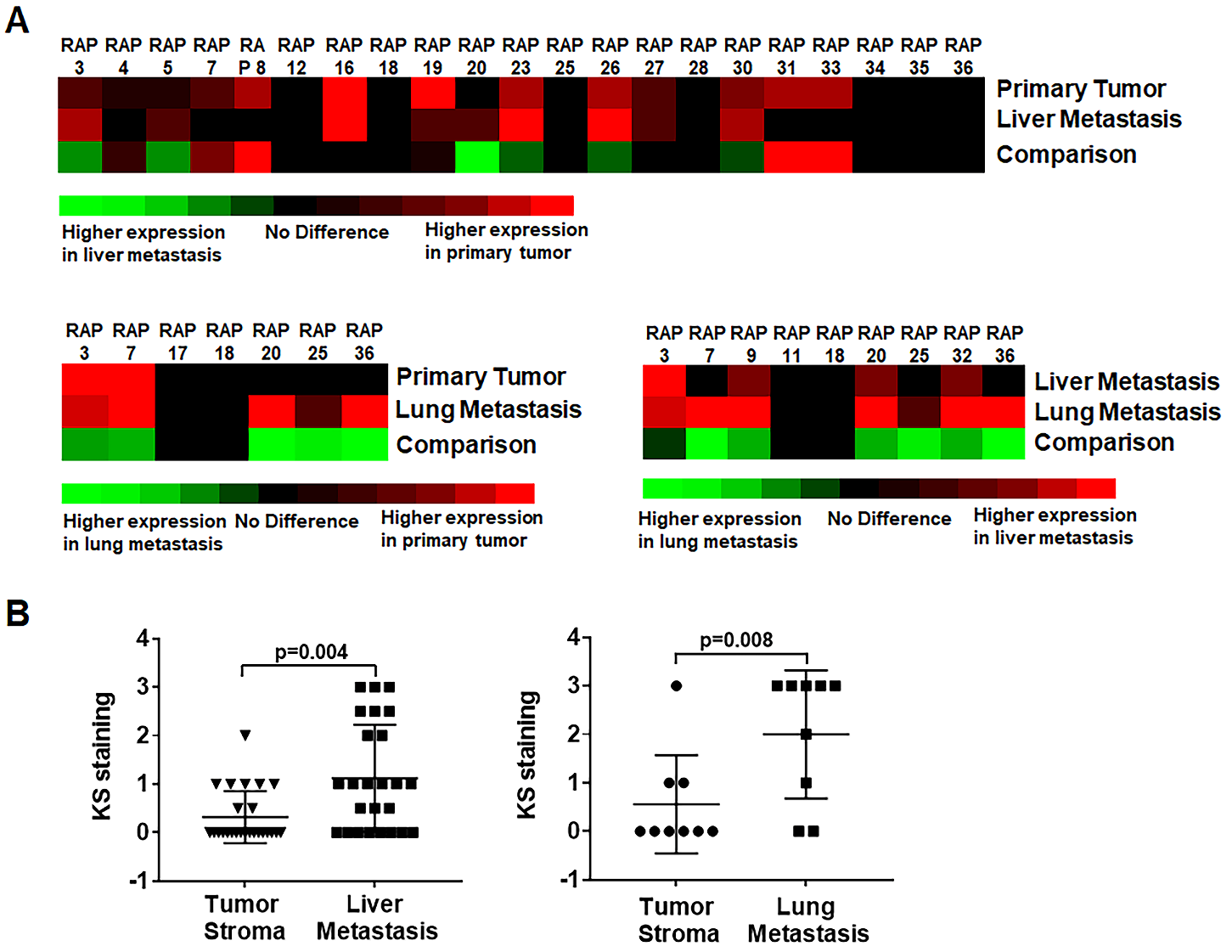


**Supplemental Figure S2. KS expression in metastatic pancreatic cancer.** (A) Heat maps show the relative expression levels of KS in primary pancreatic tumors, liver and lung metastatic tissues in a subset of 21 patients. (B) Analysis of KS staining in liver and lung metastatic tumor tissues. A p value less than 0.05 considered statistically significant
